# P-876. Factors Influencing Outcome in Patients Admitted with Melioidosis- A Single Centre Retrospective Study

**DOI:** 10.1093/ofid/ofae631.1067

**Published:** 2025-01-29

**Authors:** Raghavendra P Desai, Koushik Ramachandra, K Vidyalakshmi, Samyuktha Srinivas

**Affiliations:** Kasturba Medical College, Mangalore, Manipal Academy of Higher Education, Mangalore, Karnataka, India; Kasturba Medical College, Mangalore, Manipal Academy of Higher Education, Mangalore, Karnataka, India; Kasturba Medical College, Mangalore, Manipal Academy of Higher Education, Mangalore, Karnataka, India; Kasturba Medical College, Mangalore, Manipal Academy of Higher Education, Mangalore, Karnataka, India

## Abstract

**Background:**

Melioidosis is largely under-reported in the Indian subcontinent. This region is believed to account for 44% of total cases, with an estimated annual incidence of 52,500 cases. This retrospective study of 50 patients aimed at determining association between factors like diabetes mellitus, hypertension, existing heart disease, lung disease, liver disease, alcohol abuse, immunosuppression, symptoms at admission to the hospital, and time from symptom onset to isolation of the bacterium, on in-hospital mortality, development of acute kidney injury (AKI), need for renal replacement therapy (RRT), intensive care unit (ICU) stay and recurrence.

**Objectives:**

- To study the risk factors influencing in-hospital mortality in patients admitted with melioidosis.
- To study the association between risk factors identified and developemenet of Acute Kidney Injury, the need for Renal Replacement Therapy, ICU stay and recurrence of the disease.

**Figure:**
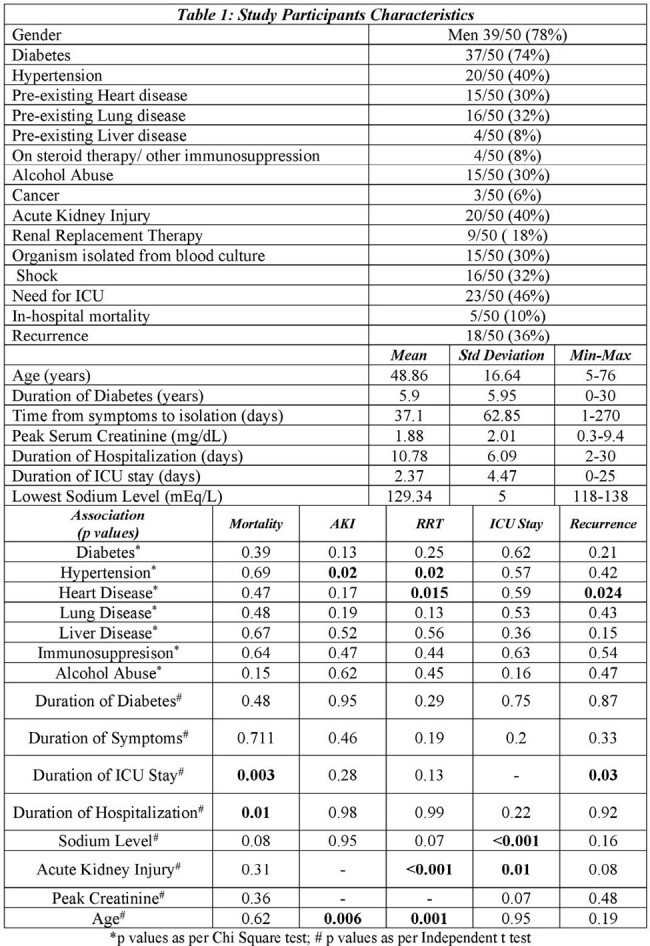

**Methods:**

Study setting: Teaching hospitals affiliated with Kasturba Medical College, Mangalore

Study design: Retrospective Cross Sectional Study

Inclusion Criteria: Patients admitted and diagnosed with Melioidosis, Age >18 years

Exclusion Criteria: Pre-existing kidney disease

**Results:**

Data analysis showed that diabetes, hypertension, pre-existing heart disease, lung disease, liver disease, alcohol abuse, immunosuppression, symptom complex, time from symptom onset to isolation of organism, and development of AKI had no significant associations with in-hospital mortality. Hypertension, pre-existing heart disease, hyponatremia, and advanced age was noted to have statistically significant associations with development of AKI. Hypertension, pre-existing heart disease, and advanced age was noted have statistically significant associations with need for RRT. Pre-existing heart disease, and longer ICU stay had statistically significant associations with recurrence of the disease. Further analysis, with respect to geographic influences is ongoing.

**Conclusion:**

In patients with Melioidosis, hypertension, pre-existing cardiac disease, hyponatremia, and advanced age were noted to have significant association with development of AKI while pre-existing cardiac disease and longer ICU stay were associated with recurrence.

**Disclosures:**

**All Authors**: No reported disclosures

